# Diagnostic Imaging of the Skeletal System: Overview of Applications in Human and Veterinary Medicine

**DOI:** 10.3390/bioengineering12121358

**Published:** 2025-12-13

**Authors:** Ana Javor, Nikola Štoković, Natalia Ivanjko, Iva Lukša, Hrvoje Capak, Zoran Vrbanac

**Affiliations:** 1 Department of Radiology, Ultrasound Diagnostics and Physical Therapy, Faculty of Veterinary Medicine, University of Zagreb, 10000 Zagreb, Croatia; ajavor@vef.unizg.hr (A.J.); ibacan@vef.unizg.hr (I.L.); hcapak@vef.unizg.hr (H.C.); zvrbanac@vef.unizg.hr (Z.V.); 2 Laboratory for Mineralized Tissues, School of Medicine, University of Zagreb, 10000 Zagreb, Croatia; natalia.ivanjko@mef.hr

**Keywords:** radiography, computed tomography, dual-energy X-ray, magnetic resonance, micro-CT, nano-CT, PET CT, quantitative ultrasound, skeletal system

## Abstract

This paper provides a comprehensive overview of the application of various radiological modalities, with a critical comparison between human and veterinary medicine. The modalities discussed include conventional radiography, dual-energy X-ray absorptiometry (DXA), computed tomography (CT), magnetic resonance imaging (MRI), ultrasound (US), quantitative ultrasound (QUS), positron emission tomography-computed tomography (PET-CT) and micro and nano computed tomography (micro-CT, nano-CT) in clinical practice and basic research of skeletal system. Radiological imaging plays a crucial role in the diagnosis, monitoring and research of skeletal system disorders in both human and veterinary medicine. In preclinical research, advanced diagnostic imaging modalities such as micro-CT and nano-CT allow for 3D quantification of trabecular and cortical bone microarchitecture for studies in bone biology, regenerative medicine and pharmacological research. Furthermore, the integration of artificial intelligence is advancing image interpretation, precision diagnostics and disease tracking. Despite their broad utility, imaging modalities must be selected based on clinical indication, species, age and anatomical region with consideration of radiation dose, cost and availability, especially in remote regions. For this reason, clinicians and radiologists remain an irreplaceable part of diagnostic imaging.

## 1. Introduction

The skeletal system forms the structural foundation of the human body, providing support, enabling movement with the muscular system while serving critical protective, hematopoietic and metabolic roles [1,2]. Due to its complex physiology and interaction with other systems, the skeleton is vulnerable to a broad spectrum of pathological processes, including traumatic injuries, degenerative diseases, infections, neoplasms and systemic metabolic disorders [3,4,5]. Timely and accurate diagnosis of these conditions is essential for guiding clinical decision-making, optimizing therapeutic strategies and improving treatment outcomes [6]. Advances in imaging technologies have greatly improved the ability to detect, characterize and monitor skeletal pathology. A range of modalities is available for both preclinical research and clinical use, each with specific principles, advantages and limitations [7]. Selection depends on the clinical context, anatomical region, suspected pathology, required diagnostic resolution as well as clinician’s decisions based on expertise [8,9]. This paper presents a comprehensive and critically comparative overview of the primary diagnostic imaging modalities used in the evaluation of skeletal pathology in both human and veterinary medicine (Table 1), providing clinicians with a systematic framework to guide the selection of the most appropriate imaging technique. It highlights their specific advantages and disadvantages in both preclinical and clinical settings, with a focus on their integration into diagnostic workflows and the emergence of hybrid imaging technologies in musculoskeletal diagnostics. One of the key aims of this work was to compare the application and availability of major diagnostic imaging modalities for skeletal pathologies between veterinary and human medicine, emphasizing similarities, differences, and their implications for clinical decision-making.

## 2. Search and Selection Strategy

A structured literature search was carried out in PubMed (MEDLINE) to retrieve publications addressing diagnostic imaging modalities and technological advancements in skeletal pathology in both human and veterinary medicine. The search combined controlled vocabulary (MeSH terms) and free-text keywords to capture both conventional and advanced imaging techniques. Specific search terms included full names and abbreviations of imaging modalities: “conventional radiography”, “dual-energy X-ray absorptiometry” (DXA), “computed tomography” (CT), “quantitative computed tomography” (QCT), “magnetic resonance imaging” (MRI), “quantitative magnetic resonance imaging” (QMRI), “ultrasound” (US), “quantitative ultrasound” (QUS), “positron emission tomography-computed tomography” (PET-CT), “micro-computed tomography” (micro-CT), and “nano-computed tomography” (nano-CT). Additional search terms targeted hybrid imaging modalities, such as “PET-MRI” and “SPECT-CT,” as well as keywords related to artificial intelligence and advanced image analysis, including “artificial intelligence,” “machine learning,” and “deep learning” in musculoskeletal and skeletal imaging contexts. Both skeletal imaging and bone imaging were used as broader search terms to ensure comprehensive literature retrieval.

Publications were included if they provided methodological insights, addressed clinical or preclinical applications, presented comparisons between imaging modalities, or reported advancements in image analysis, including AI-assisted interpretation and quantitative techniques. Both human and veterinary studies were included, with attention to comparative aspects and translational relevance across species. Only peer-reviewed articles published between 2010 and 2025 were considered to encompass foundational work and recent technological developments in skeletal imaging. Publications unrelated to imaging, case reports lacking methodological relevance, and conference abstracts without full text were excluded. To ensure comprehensive coverage, the reference lists of selected articles were also screened to identify additional relevant publications on conventional, advanced, hybrid, and AI-integrated imaging approaches in skeletal research and clinical practice.

## 3. Diagnostic Imaging Modalities

### 3.1. Conventional Radiography

**Figure 1:**
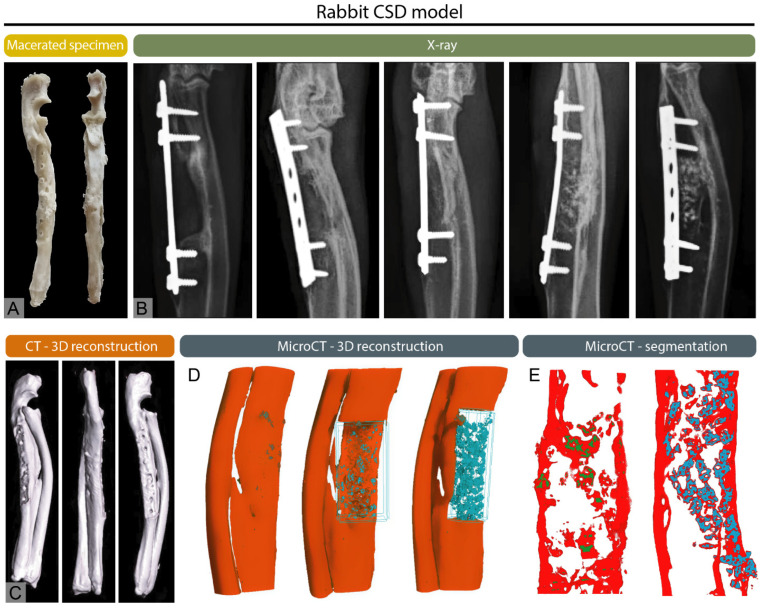
Multimodal imaging of a healed critical-sized segmental defect (CSD) of the rabbit ulna, modified from Stokovic et al. [17] (**A**) Macerated ulna showing the healed defect region. (**B**) X-rays of experimental groups illustrating variable degrees of healing, ranging from incomplete bridging to full cortical restoration. (**C**) CT 3D reconstruction, (**D**) micro-CT 3D reconstruction, and (**E**) micro-CT segmentation demonstrating regenerated bone.

**Figure 2:**
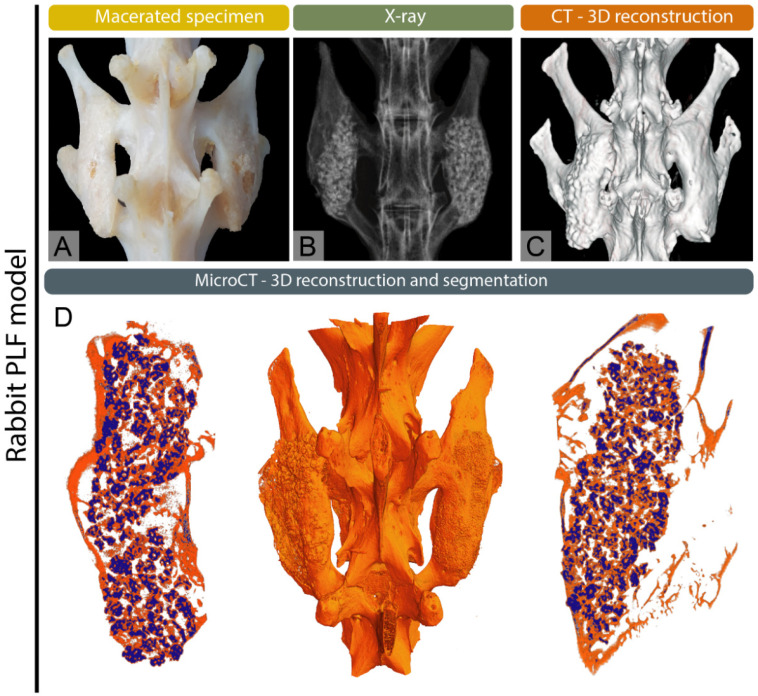
Multimodal imaging of complete postero-lateral spinal fusion (PLF) in the rabbit model, modified from Stokovic et al. [19] (**A**) Macerated spine, (**B**) X-ray image, and (**C**) 3D CT reconstruction showing fusion between adjacent transverse processes. (**D**) Micro-CT 3D reconstruction and segmentation illustrating osseointegration of newly formed bone with native transverse processes.

**Figure 3:**
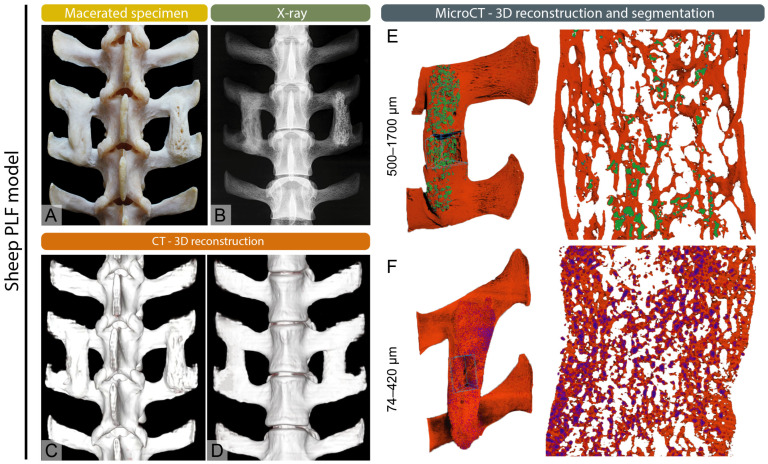
Multimodal imaging of complete postero-lateral spinal fusion (PLF) in the sheep model, modified from Ivanjko et al. [18] (**A**) Macerated spine, (**B**) X-ray image, and (**C**,**D**) 3D CT reconstructions showing continuous bilateral fusion of adjacent transverse processes. (**E**,**F**) Micro-CT reconstructions and segmentation depicting detailed cortical and trabecular architecture in the fused area.

X-ray imaging discovered by Wilhelm Conrad Roentgen, quickly became a basic tool in medical diagnostics [10,11]. Initially used to record bone fractures, X-rays today enable a wide range of diagnostic applications in both human and veterinary medicine, including the diagnosis of respiratory diseases, joint and bone pathologies, as well as the detection of tumors, foreign bodies and other indications [1,10,12]. In veterinary medicine, X-ray imaging can have some species-specific applications such as sex determination in budgerigars and reptiles [13,14]. This wide range of applications underscores the versatility and essential role of X-rays as a first-line imaging modality across species. In clinical practice, the advantages of X-ray imaging include low radiation dose, fast imaging time, and relative accessibility and cost-effectiveness [15,16]. Radiological methods play a crucial role in clinical practice and basic research [17,18,19]. They are essential for detecting fractures by determining their type, location and severity, which helps guide treatment decisions such as casting, surgical intervention or monitoring healing [20]. For precise interpretation radiological scales such as Salter-Harris, Weber scale, AO scale (Arbeitsgemeinschaft für Osteosynthesefragen) and Schatzker classification are frequently used by radiologists to give a systemic approach and unify the medical literature. Fracture classification systems provide standardized frameworks for diagnosis and treatment. The Salter–Harris classification addresses physeal fractures, the Weber scale categorizes ankle fractures by their relation to the syndesmosis, the AO scale offers a comprehensive scheme for fractures across the skeleton and the Schatzker classification defines tibial plateau fractures by pattern and articular involvement [21,22,23,24]. In veterinary medicine the scales are commonly adapted from the human medicine literature, while some are specific such as Fédération Cynologique Internationale (FCI) hip dysplasia classification [25]. Radiography is also widely used to diagnose bone deformities and conditions such as osteoarthritis, scoliosis, and congenital abnormalities, providing clear views of bone alignment, and structural deformities [26,27,28]. Additionally, they aid in screening for changes in bone density, which can indicate osteoporosis [29,30]. Radiological imaging is also valuable in identifying bone infections and detecting bone tumors, both benign and malignant [31]. Malignant tumors and infections may appear similar on X-rays with osteolytic and osteoproductive lesions visible, requiring further diagnostic procedures, such as biopsy for conformation and determination of the origin of lesions [32]. Furthermore, X-ray imaging is crucial for preoperative planning and postoperative monitoring, ensuring proper surgical outcomes and assessing bone healing (Figure 1, Figure 2 and Figure 3) [33]. For research purposes in bone healing, there are many scales that help to quantify and objectify bone healing such as the REBORNE scale or the Cook scale. The Cook and REBORNE scale are a semi-quantitative radiographic scoring system used to assess bone healing. They are used for grading fracture repair based on callus formation, cortical bridging and mineralization, ranging from non-union to complete radiographic union [34,35]. Although very useful, X-ray imaging also has limitations, such as displaying only two-dimensional images, which can make it difficult to interpret complex injuries or pathological changes. X-ray also has insufficient precision in the display of soft tissues, which is why it is often used in combination with other diagnostic methods such as ultrasound or MRI [36,37]. X-ray technology has several important applications beyond human and veterinary medicine in various industries and scientific fields such as art, archeology, material science and security screening [38]. In the last decade, there has been an effort in the radiology community to improve the image quality and provide the lowest radiation dose known as the As Low As Reasonably Achievable (ALARA) principle of imaging states [1,39]. Nevertheless, choosing X-ray imaging over more advanced modalities requires a critical assessment of the clinical question, as its inherent limitations in soft-tissue contrast, early lesion detection, and three-dimensional evaluation may lead to underdiagnosis or mischaracterization of pathology.

### 3.2. Dual-Energy X-Ray Absorptiometry

Dual-energy X-ray absorptiometry (DXA) is a widely used and highly reliable imaging technique in human medicine for measuring bone mineral density (BMD) and assessing overall bone health. It is used in veterinary medicine, but mainly in research and less commonly in clinical practice. The primary use is in the diagnosis and management of osteoporosis and the evaluation of fracture risk [40]. DXA was developed in the late 1980s and has significantly advanced bone density measurement by employing two X-ray beams at different energy levels [41]. This dual-energy approach allows for effective and precise differentiation between bones and soft tissue. This approach offers a more precise, consistent and low-radiation alternative compared to earlier methods such as single-energy X-ray absorptiometry [1,42]. The results are calculated by measuring the degree of X-ray absorption by both bone and surrounding soft tissues. They are then expressed in grams per square centimeter (g/cm^2^), providing a standardized measurement that can be compared to normative reference data [43,44]. Over time, DXA has become the gold standard in clinical practice for bone density assessment due to its high degree of accuracy, reproducibility and non-invasive nature [45,46]. One of the main advantages of DXA is its simplicity and speed. The procedure is usually completed in under 15 min and exposes patients to a minimal amount of radiation, which makes it suitable and safe for repeated assessments [47]. Even though DXA has an important role in skeletal imaging, this modality also has some limitations. It primarily focuses on one parameter, bone mineral density, and overlooks other factors like bone quality and microarchitecture of the bone. Another limitation is that DXA scans can give inaccurate results in patients with obesity. Additionally, DXA uses low-dose ionizing radiation, which can be a concern for pregnant women and children [47]. A commonly used score to assess bone health is T-score which compares a patient’s BMD to the average BMD of a healthy young adult of the same sex, while the Z-score compares the BMD to age, sex and ethnicity matched reference populations to assess whether bone loss may be due to secondary causes [48]. Moreover, DXA typically measures bone density at specific skeletal sites, such as the lumbar spine, proximal femur (hip) and sometimes the distal radius [49,50,51]. This targeted approach, while clinically relevant, may fail to detect early bone loss or subtle changes in bone quality that occur elsewhere in the skeleton. It is also important to note that certain factors can influence DXA results, potentially affecting their accuracy. Degenerative changes in the spine can lead to artificially elevated BMD measurements, masking underlying bone loss and complicating interpretation of the data [52,53,54,55]. Additionally, artifacts from surgical implants or improper patient positioning during the scan may further distort results [56]. Nevertheless, DXA remains a valuable tool in both clinical and research settings and is emerging as a valuable tool in veterinary medicine. Accordingly, clinicians should base the decision to use DXA on a critical appraisal of its ability to provide accurate site-specific BMD measurements while recognizing its limitations in assessing bone quality and susceptibility to artifacts, ensuring that DXA findings are interpreted within the broader clinical and diagnostic context.

### 3.3. Computed Tomography

Computed tomography (CT) was discovered in the early 1970s as groundbreaking imaging technology by Godfrey Hounsfield and Allan Cormack. The Hounsfield unit quantifies the attenuation of X-ray beams by tissues in a CT scan, using water and air as reference points [57,58]. Advanced X-ray technology and computer algorithms are used for processing using mathematical algorithms to reconstruct images slice by slice, which can then be rendered into three-dimensional models [1,57]. Modern CT systems, such as multislice or multidetector CT (MDCT) capture multiple slices simultaneously allowing for faster imaging and higher spatial resolution. Helical (spiral) CT, introduced in the 1990s, further improved imaging by enabling continuous data acquisition as the patient moves through the scanner [59]. CT provides high-resolution visualization of bone, making it invaluable for assessing complex, subtle, or occult fractures and for surgical planning [60]. It is also used to evaluate bone tumors, assess osteoporosis-related bone density, and identify degenerative joint changes [61,62]. Additionally, CT helps detect infections and assess spinal disorders such as disc herniation and spinal stenosis [63,64]. Three-dimensional reconstructions offer detailed anatomical views important for orthopedic and spinal procedures (Figure 1, Figure 2 and Figure 3). In veterinary medicine, CT has become a routine diagnostic tool for small and large animals over the past decade, and although highly effective in skeletal diagnostics, it is often combined with MRI or ultrasound for comprehensive soft-tissue evaluation [65,66,67,68]. Modern CT scanners are fast, enabling imaging in emergencies in both human and veterinary medicine [69,70]. In recent years there has been a lot of debate about the overuse of CT imaging in emergency cases, but nevertheless it remains an invaluable tool because of its speed and the amount of clinical information received [71]. However, CT also has some disadvantages as it exposes patients to higher doses of ionizing radiation compared to traditional X-rays which can be a concern with repeated use [72]. CT scans are also costly, limiting accessibility for some patients. In veterinary medicine, the size of the gantry and table load limits can restrict imaging of large animals. While excellent for bone imaging, CT is less effective than MRI in evaluating soft tissue structures, such as cartilage and ligaments. Metal implants may cause artifacts that obscure images [73]. Additionally, the use of contrast agents in some studies can pose risks of allergic reactions or other adverse effects [1,68]. However, the decision to employ CT imaging in skeletal diagnostics and research should be guided by its primary indications such as complex or occult fractures, bone tumor evaluation, and preoperative planning where it offers clear advantages over conventional X-rays, while carefully considering its limitations and ensuring its use is justified by the specific clinical or research objectives.

### 3.4. Quantitative Computed Tomography

Quantitative Computed Tomography (QCT) is an advanced imaging technique used to measure bone mineral density with high precision [74,75]. QCT offers volumetric measurements, allowing for a more accurate assessment of bone quality and fracture risk [76]. The technique is particularly useful in evaluating trabecular bone, which is more metabolically active and susceptible to osteoporotic changes. QCT is commonly applied to the lumbar spine, hip, and forearm, making it valuable in osteoporosis diagnosis and monitoring, as well as in research settings studying metabolic bone diseases [74]. Clinically, it is used to assess fracture risk, guide treatment decisions and evaluate the effects of osteoporosis therapies [44]. Traditional phantom-based QCT requires the use of an external calibration phantom, scanned simultaneously with the patient, to convert Hounsfield units into absolute BMD values [77]. This approach ensures high precision and reproducibility across scanners and time points. In contrast, phantom-less QCT has emerged as an alternative method, particularly in opportunistic screening, where CT scans originally acquired for other purposes are retrospectively analyzed. Phantom-less QCT estimates BMD by referencing internal tissues such as muscle and fat for calibration [74,77]. The advantages of QCT include its ability to differentiate cortical from trabecular bone and its independence from body size or soft tissue artifacts. However, disadvantages include higher radiation exposure compared to DXA, greater cost and less widespread availability [78,79].

### 3.5. Micro-Computed Tomography

Micro-computed tomography (micro-CT) is a vital tool in skeletal research, providing high-resolution, three-dimensional imaging to study bone structure and function at the microstructural level [80]. It allows for detailed visualization of cortical and trabecular bone and precise measurements of bone mineral density, volume, and connectivity (Figure 1, Figure 2 and Figure 3). Micro-CT is widely used to investigate bone diseases like osteoporosis, osteoarthritis, and neoplastic disease [27,81,82]. This diagnostic tool has an important role in preclinical trials, and it plays a key role in studying bone growth, remodeling, and evaluating therapies, such as drug treatments and implants [83,84,85,86]. Although micro-CT offers non-destructive imaging and high spatial resolution, it requires specialized equipment and expertise and involves increased radiation exposure. Despite these challenges, micro-CT is essential in advancing skeletal research [87,88,89,90]. Micro-CT applications extend beyond preclinical investigations as these techniques are also valuable for the microstructural analysis of excised human bone specimens [91]. The most advanced type of micro-CT imaging is using spectral micro-CT. This technology uses an advanced imaging technique that enhances traditional micro-CT by capturing multiple energy levels, allowing for the differentiation of materials based on their specific attenuation properties. This technique provides more detailed and precise information about the composition and structure of tissues or materials at the microstructural level [92,93,94].

### 3.6. Nano-Computed Tomography

Nano-computed tomography (nano-CT) takes imaging to the nanoscale, offering more detailed views of bone structure. This allows visualization of the bone matrix, mineralization, analysis of bone for fine-scale architecture and material quality, study of synthetic bone scaffolds for orthopedic research and the imaging of vascular networks [95]. Such high resolution is crucial for studying bone diseases like osteoporosis, osteogenesis imperfecta and bone cancer at the cellular level [96,97]. In skeletal research, nano-CT plays an important role in understanding how bones remodel, evaluating bone quality, and testing how different treatments or therapies affect bone healing and regeneration. It is also a powerful tool for assessing biomaterials and bone implants, giving a closer look at how they integrate with bone tissue [98]. Despite its advantages, nano-CT has several limitations. Along with high costs, it cannot fully replace histological assessment, especially when specific staining is needed for cellular analysis [99]. Optimizing parameters often require pilot experiments. While nano-CT offers higher resolution than micro-CT, its smaller sample size and limited volume of interest may impact the reliability of quantitative data [100,101].

### 3.7. Positron Emission Tomography-Computed Tomography

Fluorodeoxyglucose (FDG) is a radiolabeled glucose analog used as a tracer in positron emission tomography computed tomography (PET-CT) scans [102]. PET-CT is currently rarely available in routine veterinary practice and is mostly confined to referral centers, university hospitals and research institutions. The principle of FDG uptake is based on the fact that malignant cells and other metabolically active tissues exhibit significantly increased glucose consumption compared to normal tissues [103]. Once administered intravenously, FDG is taken up by these tissues but is not fully metabolized. FDG-PET/CT is widely used in oncology for cancer diagnosis, staging, metastasis detection, and monitoring therapy or recurrence [104,105]. It also has important roles beyond oncology: in neurology, it helps identify neurodegenerative diseases by detecting characteristic temporoparietal hypometabolism [106]. In cardiology, it is used to assess myocardial viability and detect cardiac inflammation [107]. The technique is also increasingly applied in the detection of active bone inflammation [108]. Beyond clinical practice, PET-CT is a valuable tool in research for studying skeletal biology. It enables non-invasive quantification of bone turnover and metabolic activity, aiding in the assessment of bone diseases and monitoring the effects of pharmacological interventions [109]. PET-CT imaging is valuable for studying therapies that target bone metabolism and for investigating the mechanisms of bone remodeling. Two commonly used tracers [^18^F]-fluorodeoxyglucose ([^18^F]-FDG) and [^18^F] sodium fluoride ([^18^F]NaF) provide complementary information on skeletal physiology. [^18^F]-FDG accumulates in regions with active bone formation, such as the epiphyseal and metaphyseal areas of long bones [110]. Its uptake correlates with osteoblastic metabolic activity and is influenced by systemic factors like insulin [111]. However, FDG is not bone-specific and is localized to other metabolically active tissues. In contrast, [^18^F]NaF is a bone-specific tracer that incorporates directly into hydroxyapatite, allowing sensitive assessment of bone perfusion, mineralization, and remodeling. It provides faster soft-tissue clearance, higher bone-to-background contrast, and better spatial resolution than FDG, making it particularly effective for detecting microdamage. These features establish [^18^F]NaF as the gold standard for bone PET imaging [112]. However, PET-CT imaging has limitations that should be acknowledged. In inflammatory and infectious diseases, FDG uptake patterns are often more diffuse and can overlap with physiological uptake, which reduces the specificity of findings [110]. For instance, normal uptake can be seen in skeletal muscle, the gastrointestinal tract, or brown adipose tissue, making interpretation challenging [113]. This non-specific uptake can lead to false-positive results and may prompt unnecessary biopsies, additional testing or unwarranted treatment, each of which carries its own risks and potential side effects. Another significant limitation involves the use of corticosteroids, which are known to suppress FDG uptake and reduce the sensitivity of PET-CT scans [113,114]. Thus, accurate interpretation of PET-CT findings is crucial, as the differing specificity of tracers, FDG being non-specific and NaF bone-targeted, can directly influence diagnostic decisions and patient management.

### 3.8. Magnetic Resonance Imaging

Magnetic resonance imaging (MRI), based on the principles of nuclear magnetic resonance (NMR), has transformed both the diagnostic and research approaches to the skeletal system [115,116]. It is widely used in both human and veterinary medicine for detailed imaging of soft tissues and skeletal structures. The availability of MRI in veterinary medicine is more limited than in human medicine, mainly due to high costs. High-field MRI (≥1.5 T) is mostly available in specialized referral centers and university hospitals, whereas low-field MRI (<1 T) is more common in general veterinary practice. Low-field MRI and high-field MRI differ in field strength, image quality, and clinical use. Low-field has lower signal-to-noise ratio and resolution, suitable for larger structures and general soft tissue imaging, while high field MRI provides higher resolution for small lesions and cartilaginous structures [1]. MRI offers high soft tissue contrast, allowing detailed visualization of bone marrow, cartilage, ligaments and surrounding soft tissues, which is superior to other imaging modalities like X-ray or CT [117]. In skeletal diagnostics, MRI excels in detecting bone marrow edema, stress fractures, and early stages of osteomyelitis or neoplastic changes [118]. However, its limitations include lower resolution for cortical bone and susceptibility to motion artifacts, as well as high operational costs and longer scan times compared to other methods. The interaction of ferromagnetic objects with strong magnetic fields is a critical patient safety consideration in MRI [118,119]. Therefore, thorough screening for ferromagnetic materials and adherence to strict safety protocols are essential to mitigate these risks and ensure patient safety during MRI procedures [118,119,120]. MRI signal characteristics depend on the interaction between magnetic forces and tissue composition. Images are generated by recording the time protons take to return to their resting state after magnetization. In T1-weighted images, fat appears bright, muscle intermediate, and water dark, making T1 useful for detecting fat deposition in chronic myopathies [121]. In contrast, T2-weighted images highlight water, edema and inflammation, making them ideal for identifying acute muscle changes. Overall, T1 is best for chronic neuromuscular disease, while T2 is superior for detecting acute or inflammatory muscle conditions [122]. Common scoring systems used in clinical practice include the Whole-Organ Magnetic Resonance Imaging Score (WORMS) for osteoarthritis, the Spondyloarthritis Research Consortium of Canada (SPARCC) score for sacroiliitis or the Rheumatoid Arthritis Magnetic Resonance Imaging Score (RAMRIS) for evaluating joint inflammation and damage [123,124,125]. These scales enable standardized assessment of disease severity and progression, facilitating both diagnosis and monitoring in clinical and research settings. Additionally, functional MRI (fMRI) and micro-MRI (µMRI) have limited but emerging applications in veterinary skeletal diagnostics. fMRI, though primarily developed for brain mapping, has little direct role in bone pathology. However, it may provide indirect insights into pain perception and neurovascular responses associated with musculoskeletal disease in patients [126]. For clinicians and researchers, MRI demonstrates its greatest strength in the evaluation of bone marrow pathology, early-stage osteomyelitis, stress fractures, cartilage integrity, and soft tissue involvement, providing critical diagnostic and research insights that are not achievable with conventional radiography or CT [127].

### 3.9. Quantitative Magnetic Resonance Imaging

Quantitative magnetic resonance imaging (QMRI) is an advanced MRI technique that provides objective, numerical measurements of tissue properties rather than just anatomical imaging [128]. In veterinary practice, QMRI is primarily used in research and specialized referral settings. Unlike conventional MRI, which produces qualitative images based on contrast differences, QMRI quantifies physical and biochemical tissue parameters, making it highly valuable for both research and clinical applications [129]. QMRI utilizes imaging parameters such as T1 and T2 relaxation times, which are influenced by the bone marrow’s water content and bone architecture, to estimate bone mineral density [130]. Advantages of this diagnostic imaging modality include objective, reproducible measurements of tissue properties, enhancing early disease detection and enabling standardized biomarkers for monitoring disease progression and treatment response [131]. Its non-invasive nature and versatility across various medical fields makes this diagnostic tool invaluable for complicated clinical cases and research. However, QMRI faces challenges such as longer scan times, increased computational demands, higher costs and the necessity for specialized technical expertise which make it unavailable for a part of the population. Additionally, variations in measurements across different MRI scanners can affect diagnostic accuracy, highlighting the need for rigorous quality control procedures to ensure consistency and reliability in clinical applications [132,133]. In skeletal diseases diagnostics it is commonly used for assessing bone, cartilage and muscle integrity. For diagnostics of muscle integrity most common detected compositional changes include fat infiltration, quantification of active muscle damage, fibrosis, microstructure assessment which include muscle cell size and intracellular/extracellular space, muscle contraction and muscle architecture [133,134,135].

### 3.10. Ultrasound

Ultrasound (US) imaging relies on high-frequency sound waves that propagate through tissue and are reflected at interfaces with differing acoustic impedance. The returning echoes are detected and converted into real-time images, allowing visualization of tissue structure and dynamics. In bone pathology, US has become a valuable, non-invasive tool in both human and veterinary medicine for clinical and research applications [136]. It is used to assess fracture healing, cortical integrity, callus formation, and early detection of osteoporosis-related changes, particularly in superficial bones. Ultrasound enables guidance during biopsies and aspirations, longitudinal monitoring of fracture healing, as well as evaluation on vascularisation [137,138]. In preclinical research, it allows longitudinal monitoring of bone regeneration and mineralization, as well as assessment of therapeutic interventions without repeated radiation exposure [139]. Although limited by depth penetration and operator dependency, US provides real-time imaging and high-resolution assessment of cortical surfaces, complementing radiography, computed tomography, and magnetic resonance imaging in comprehensive skeletal evaluation [140]. Clinicians and researchers should interpret ultrasound findings in the context of complementary imaging modalities, as its sensitivity and specificity for detecting subtle or deep bone pathology remain limited, emphasizing the need for standardized protocols and operator training to maximize diagnostic reliability.

### 3.11. Quantitative Ultrasound

The introduction of pulse Doppler allowed for a quantitative blood flow assessment. With full digitalization quantitative ultrasound (QUS) techniques emerged, utilizing the physical properties of sound waves for diagnostic purposes [141,142]. Quantitative ultrasound is a non-invasive imaging technique used to assess skeletal health, particularly for evaluating bone mineral density [143]. Sound waves are utilized to estimate the mechanical properties of bone, including density and elasticity, without exposing the patient to ionizing radiation. QUS measures the distance and velocity at which sound waves travel from the source to the detector [144]. Ultrasound waves experience significant reflection and attenuation when encountering air or gas, limiting their effectiveness in imaging areas with substantial air content, such as the lungs and intestines [145]. As a result, QUS is typically performed at peripheral sites with minimal soft tissue, with the calcaneus being the most used measurement site in human medicine [146]. In veterinary medicine QUS was used in research in measuring the speed of sound measurements in axial transmission mode used to precisely measure superficial cortical bone properties of third metacarpal bone, radius, and tibia in horses [147]. Although some publications are present about the use of QUS in both dogs and horses, this modality is not well-established and routinely used in everyday veterinary practice. Its broader adoption in veterinary medicine is further limited by the scarcity of species-specific calibration standards, limited commercial availability of dedicated veterinary QUS devices, and ongoing challenges in validating its diagnostic accuracy across different skeletal sites and animal sizes [148,149]. Quantitative ultrasound produces two key variables: the speed of sound (SOS) and broadband ultrasound attenuation (BUA). SOS reflects the velocity at which sound waves travel through tissue, while BUA measures the degree of wave attenuation, expressed in decibels per megahertz (dB/MHz) [150]. The wave’s oscillation and the rate at which its intensity diminishes over time are expressed as BUA. The energy loss per unit of time is influenced by both bone mineral density and bone architecture, providing insight into the quantity and density of cortical and trabecular bone [151]. This diagnostic imaging modality is used in clinical settings to assess bone health and evaluate the risk of osteoporosis and fractures. The advantages of QUS are that it is a valuable, radiation-free tool for bone health assessment, suitable for repeated use in sensitive populations like children and pregnant women [152]. It is portable and cost-effective. Some of the disadvantages in clinical practice are that it primarily measures peripheral bones like the calcaneus, which may not represent central sites such as the spine or hip, where osteoporotic fractures are most common [153,154]. QUS is less precise than DXA, with significant variability between devices and operators. Itis less reliable in obese patients due to soft tissue interference. While useful for screening, QUS is complementary to DXA and not the gold standard for diagnosing osteoporosis [44,155]. Advancement of quantitative ultrasound in both human and veterinary medicine necessitates systematic studies to refine calibration methods, standardize measurement protocols, and validate its parameters across species and skeletal sites, ensuring consistent and reproducible outcomes for research and clinical practice.

**Table 1:** Imaging modalities in skeletal pathology and their diagnostic roles.

Imaging Modality	Indications	Contraindications	Advantages	Disadvantages	References
X-ray imaging	Fracture, joint dislocation, bone deformities, degenerative joint/spine disease, osteoporosis screening, tumors, pre/postoperative monitoring	Pregnancy, contrast allergy, severe obesity	High spatial resolution for bone, fast, accessible, portable	Limited soft tissue contrast, 2D only, ionizing radiation	[1,12,15,16,26,27,28,29,31,32,33,36,37,39,64]
Dual-energy X-ray absorptiometry (DXA)	Osteoporosis diagnosis, fracture risk, BMD monitoring, lean mass/fat distribution	Pregnancy, severe obesity, recent contrast, inability to remain still	High precision BMD, low radiation, non-invasive standard	Limited microarchitecture info, artifacts affect accuracy	[1,40,41,44,45,46,47,48,49,50,52,54,55,56,78]
Computed Tomography (CT)	Complex fractures, degenerative diseases, tumor and infection assessment, 3D surgical planning	Pregnancy, contrast allergy, severe obesity	Three-dimensional bone imaging, fast acquisition, detailed fracture visualization	Higher radiation dose, limited soft tissue contrast	[1,62,63,64,67,68,69,70,72,73,74,85,117]
Quantitative Computed Tomography (QCT)	Volumetric BMD, osteoporosis progression, patients unsuitable for DXA, opportunistic screening	Pregnancy, contrast allergy, severe obesity	Volumetric BMD, distinguishes cortical/trabecular bone, sensitive	Higher radiation, cost, calibration required, limited availability	[1,75,77,78,79]
Micro-CT	Preclinical bone microstructure analysis, research	None reported	High-resolution 3D bone microarchitecture, quantitative analysis	Limited to small samples, high radiation, costly	[1,80,81,82,83,84,85,86,87,88,89,90,91,92,93,94,98]
Nano-CT	Bone ultrastructure research, biomaterials, tissue engineering	None reported	Nanoscale resolution, quantifies nano-architecture	Small sample size, expensive, technical complexity	[95,96,97,98,99,101]
Positron emission tomography-computed tomography (PET-CT)	Cancer detection/staging, neuro/cardiac/inflammatory disease assessment	Pregnancy, radiotracer allergy, metal implants, severe obesity	Detects metabolic changes early, whole-body imaging, quantitative uptake	High cost, ionizing radiation, limited spatial resolution, limited specificity	[102,103,104,105,106,107,108,109,110,111,112,113,114,156]
Magnetic resonance imaging (MRI)	Soft tissue injury, bone marrow edema, tumor staging, congenital/inflammatory disorders	Metallic implants, obesity, allergy, pregnancy, claustrophobia	Superior soft tissue contrast, multiplanar imaging, no radiation	High cost, sensitive to motion, limited cortical bone imaging	[115,116,117,118,119,120]
Quantitative MRI (QMRI)	Cartilage and trabecular bone quantification, marrow composition, early OA/osteoporosis detection	Same as MRI	Objective tissue quantification, serial monitoring, no radiation	Technical complexity, long scan/analysis time, variable reproducibility	[128,129,130,131,135]
Ultrasound (US)	Fractures, periosteal reactions, synovitis, soft tissue, procedural guidance	None major	Real-time, portable, high resolution superficial imaging	Limited penetration, operator dependent, limited bone imaging	[136,137,138,139,140]
Quantitative ultrasound (QUS)	Osteoporosis screening, bone quality, peripheral fracture risk	Acute injury/amputation site, obesity, recent surgery	Radiation-free, portable, low cost, good for mass screening	Limited to peripheral sites, less accurate than DXA/QCT	[1,46,54,142,143,144,145,146,147,148,149,150,151,152,153]

## 4. Key Differences and Selection of Imaging Methods in Human and Veterinary Medicine

Imaging modalities are used daily in both human and veterinary practice, but both fields have their own unique challenges in clinical application, patient handling, technical approach and post processing and interpretation of the images. The clinical applications of radiological techniques often overlap between human and veterinary medicine, for example, in the diagnosis of fractures, tumors, and joint diseases. However, veterinary medicine also involves diagnostic applications that are specific to animals, such as sex determination, assessment of lameness and breeding-related examinations [13,14,157,158]. A key distinction in the application of radiological methods between human and veterinary medicine lies in the fact that veterinary medicine encompasses a wide variety of animal species, which differ significantly in terms of size, anatomy, and physiology. These differences have important practical implications. Consequently, veterinary radiology requires adaptations and specialized approaches to accommodate the diverse anatomical and physiological characteristics of different species. These species-dependent requirements are further compounded by practical limitations such as gantry diameter and table load constraints, challenges in imaging large animal species, the need for adapted acquisition protocols, and careful consideration of field-of-view and motion-management strategies. On the other hand, certain radiological techniques used primarily for research purposes, such as micro-CT and nano-CT are inherently limited by their relatively small field of view. These modalities can be used for in vivo imaging only in small animals, such as mice and rats. In contrast, bone samples from larger animals or humans can generally be imaged with these techniques only post-mortem or from a bone sample [80,81,82]. Furthermore, patient handling in veterinary medicine is often more challenging, frequently necessitating the use of appropriate restraint techniques as well as sedation or anesthesia [159]. Given this, close collaboration with anesthesiologists is essential, as they must carefully tailor anesthesia protocols to the specific species, size, and health status of each animal to ensure both safety and optimal imaging conditions. Additionally, in some cases, radiologists or radiology technicians must be present alongside the animal to ensure proper positioning during imaging. This requirement increases the potential for occupational radiation exposure among radiology personnel [160,161,162]. Such considerations underscore the importance of implementing strict radiation safety protocols and protective measures in veterinary imaging settings. An important distinction between veterinary and human medicine lies in the availability of radiological equipment. In large animal veterinary practice, it is often impossible or economically impractical to transport the animal to a clinical center equipped with advanced imaging modalities. As a result, there is a significant reliance on portable radiography within veterinary medicine [162]. It is also important to note that in certain countries or regions, access to radiological techniques in veterinary medicine is more limited compared to human medicine. Additionally, in some cases, available imaging methods may be economically inaccessible or cost-prohibitive for animal owners, especially for advanced imaging of large animals. These factors underscore the unique logistical and financial challenges associated with radiological diagnostics in veterinary practice. In human medicine, imaging protocols are highly standardized, and radiologists are often assisted by artificial intelligence [163,164]. In contrast, veterinary medicine is less standardized, and image interpretation frequently requires detailed knowledge of species-specific anatomy and breed variations [165]. Consequently, veterinary radiologists must possess a comprehensive understanding of anatomy and pathology across multiple species, which demands a broader scope of expertise. Moreover, the organization of work in many veterinary clinical centers often limits the opportunity for subspecialization in specific imaging modalities or anatomical regions, a practice that is common in human medical centers. The systematic selection of imaging modalities in skeletal diagnostics and research requires careful consideration of their complementary strengths, clinical relevance and species-specific applicability [32,70,71]. Conventional radiography remains the cornerstone for initial evaluation of fractures and degenerative changes, often complemented by CT for complex anatomical regions and MRI for assessing bone marrow, cartilage, and soft tissue involvement [166]. DXA is widely applied for the clinical assessment of osteoporosis, whereas QUS is used as a screening tool but less frequently in routine practice. PET-CT plays a critical role in oncology and inflammatory bone disease by combining morphological and metabolic information. In contrast, veterinary medicine relies heavily on radiography as the primary diagnostic tool, with ultrasound frequently employed for musculoskeletal soft tissue assessment and guided interventions [134]. Advanced modalities, including CT and MRI, are increasingly employed in referral and research settings to provide a detailed characterization of skeletal pathology in companion animals [118]. Nevertheless, when applying CT, MRI and other similarly advanced radiological techniques in veterinary medicine, the limitations previously described must be considered.

## 5. Artificial Intelligence in Diagnostic Imaging

Artificial intelligence (AI) has become an integral component of diagnostic imaging in human medicine, where large, standardized datasets and well-established imaging protocols enable the development of robust algorithms for automated image interpretation, lesion detection, segmentation, and workflow optimization [167]. These tools have demonstrated measurable improvements in diagnostic accuracy, consistency, and reporting efficiency across modalities such as radiography, CT, and MRI [164]. In contrast, the application of AI in veterinary diagnostic imaging remains in an earlier developmental stage. Species heterogeneity, wide anatomical variation, smaller and less standardized datasets, and greater variability in imaging protocols pose significant challenges to algorithm training and validation [163,168]. Despite these limitations, emerging veterinary AI tools particularly for radiography and CT are beginning to support clinicians through automated measurements, pattern recognition, and triage functions [169]. As data availability and standardization improve, and as cross-species transfer learning methods advance, the gap between human and veterinary AI applications is expected to narrow, enhancing diagnostic accuracy, facilitating evidence-based decision-making, and supporting translational research across both fields. In skeletal imaging, AI has already demonstrated promise in tasks such as automated fracture detection on radiographs, segmentation and quantification of trabecular and cortical bone in CT or micro-CT scans, and assessment of bone morphology and density in DXA and QCT studies. In veterinary medicine, AI is increasingly being applied to automated gait and posture analysis. A notable limitation in AI-assisted diagnostic imaging, particularly pronounced in veterinary practice, is the susceptibility of algorithms to positional artifacts. In human imaging, standardized patient positioning and uniform imaging protocols help minimize variability, allowing AI models to interpret anatomical structures and skeletal lesions with high reliability. In contrast, veterinary patients often require manual restraint, sedation, or anesthesia, and their diverse body conformations make consistent positioning more difficult, which can reduce the accuracy of AI-based skeletal assessments [170]. As a result, images may contain rotation, foreshortening, or asymmetry that can mislead AI algorithms trained on limited or idealized datasets. These positional artifacts can reduce diagnostic accuracy, impair automated landmark detection, and increase the likelihood of false positives or negatives. Addressing this challenge will require the development of more robust, species-inclusive training datasets and algorithmic approaches capable of accommodating greater anatomical and positional variability [171].

## 6. Conclusions

Diagnostic imaging of the skeletal system is essential in both human and veterinary medicine, but the two fields face distinct challenges. Accurate detection and assessment of skeletal pathologies depend on the careful selection of appropriate diagnostic imaging modalities in both clinical and preclinical settings [8,156,172,173]. Conventional radiography remains the first-line tool for detecting fractures and gross skeletal abnormalities in both humans and animals, while CT is preferred for complex bony structures, such as the spine or skull and for preoperative planning [15,16,174]. MRI excels in soft tissue evaluation, including tendons, ligaments, cartilage, and muscle and is particularly valuable in pediatric patients where radiation exposure must be minimized [29,175,176,177]. DXA is the gold standard for assessing bone mineral density and osteoporosis, whereas QUS can serve as a rapid screening tool [46,146,147,148]. PET-CT provides combined metabolic and structural information, making it ideal for oncologic and inflammatory bone disease. Its high cost makes it largely unavailable in veterinary medicine clinical applications [178]. In veterinary medicine, radiography is the mainstay, especially for large animals where portability and cost are limiting factors, while CT and MRI are increasingly used in companion animals for detailed characterization of skeletal pathology. Effective skeletal diagnostics thus relies on multimodal approaches, selected according to the specific tissue of interest, patient size, clinical context and available resources. Hybrid imaging modalities, such as PET/MRI and SPECT/CT, further expand the diagnostic capabilities by combining functional or metabolic imaging with high-resolution anatomical detail from MRI or CT. These hybrid approaches offer improved soft-tissue contrast (PET/MRI) or enhanced bone imaging with lower radiation exposure (SPECT/CT), enabling more comprehensive evaluation of complex skeletal pathologies in both research and clinical settings [1,61]. In preclinical studies, imaging small animals requires high-resolution systems and specialized protocols while respecting the 3R principle (Replacement, Reduction, Refinement) [179,180,181,182]. The integration of anatomical and functional imaging continues to advance diagnostic accuracy and translational applications, ultimately improving outcomes in both human and veterinary skeletal medicine. In human radiology, artificial intelligence (AI) is increasingly integrated into standardized workflows, assisting with image interpretation, lesion detection and workflow optimization to improve diagnostic accuracy and efficiency [167,168]. In veterinary radiology, AI applications are still emerging, with challenges related to species diversity, limited training datasets, and variable imaging protocols, but they are useful for supporting clinicians in routine diagnostics and research [163,164]. Complementary non-imaging methods, such as vibroelastography, can work synergistically with these modalities by providing additional mechanical and functional information, further improving diagnostic precision. The integration of anatomical and functional imaging continues to advance diagnostic accuracy and translational applications, ultimately improving outcomes in both human and veterinary skeletal medicine [183].

## Data Availability

No new data were created or analyzed in this study.

## Abbreviations

DXA	Dual-energy X-ray absorptiometry
CT	Computed tomography
MRI	Magnetic resonance imaging
QUS	Quantitative ultrasound
PET-CT	Positron emission tomography-computed tomography
QCT	Quantitative computed tomography
micro-CT	Micro-computed tomography
nano-CT	Nano-computed tomography
BMD	Bone mineral density
MDCT	Multidetector computed tomography
ALARA	As Low As Reasonably Achievable
FDG	Fluorodeoxyglucose
[^18^F]-FDG	Fluorine-18 fluorodeoxyglucose
[^18^F]	sodium fluoride (NaF)
NMR	Nuclear magnetic resonance
WORMS	Whole-organ magnetic resonance imaging score
SPARCC	Spondyloarthritis Research Consortium of Canada
RAMRIS	Rheumatoid Arthritis Magnetic Resonance Imaging Score
fMRI	functional MRI
QMRI	Quantitative magnetic resonance imaging
US	Ultrasound
SOS	Speed of sound
BUA	Broadband ultrasound attenuation
CSD	Critical segmental defect
PLF	Postero-lateral fusion
3R	replacement, reduction, refinement
AI	artificial intelligence
